# Establishment of normative ranges of the healthy human immune system with comprehensive polychromatic flow cytometry profiling

**DOI:** 10.1371/journal.pone.0225512

**Published:** 2019-12-11

**Authors:** John S. Yi, Marilyn Rosa-Bray, Janet Staats, Pearl Zakroysky, Cliburn Chan, Melissa A. Russo, Chelsae Dumbauld, Scott White, Todd Gierman, Kent J. Weinhold, Jeffrey T. Guptill

**Affiliations:** 1 Department of Surgery, Duke University School of Medicine, Durham, NC, United States of America; 2 Biomat USA–Grifols Plasma Operations, United States of America; 3 Duke Clinical Research Institute, Durham, NC, United States of America; 4 Department of Biostatistics and Bioinformatics, Duke University School of Medicine, Durham, NC, United States of America; 5 Department of Neurology, Duke University School of Medicine, Durham, NC, United States of America; Jackson Laboratory, UNITED STATES

## Abstract

Existing normative flow cytometry data have several limitations including small sample sizes, incompletely described study populations, variable flow cytometry methodology, and limited depth for defining lymphocyte subpopulations. To overcome these issues, we defined high-dimensional flow cytometry reference ranges for the healthy human immune system using Human Immunology Project Consortium methodologies after carefully screening 127 subjects deemed healthy through clinical and laboratory testing. We enrolled subjects in the following age cohorts: 18–29 years, 30–39, 40–49, and 50–66 and enrolled cohorts to ensure an even gender distribution and at least 30% non-Caucasians. From peripheral blood mononuclear cells, flow cytometry reference ranges were defined for >50 immune subsets including T-cell (activation, maturation, T follicular helper and regulatory T cell), B-cell, and innate cells. We also developed a web tool for visualization of the dataset and download of raw data. This dataset provides the immunology community with a resource to compare and extract data from rigorously characterized healthy subjects across age groups, gender and race.

## Introduction

Flow cytometry is a powerful tool for investigating the cellular components of the immune system. This technique can quantitate a steadily increasing number of cell parameters simultaneously and is capable of defining the phenotype and function of cell subsets, even in rare cell populations. The identification of immune subsets in the blood is an important diagnostic and monitoring tool in the clinic for certain immunological and non-immunological conditions, including but not limited to, stem cell transplantation, vaccine development, cancer immunotherapy, hereditary immunodeficiency disorders, autoimmune conditions, and monitoring of CD4 T cells in HIV patients.

A limitation of flow cytometry is the absence of comprehensive normative data that has kept up with the advances in our understanding of immunologic cell subsets in humans. Reference values for basic lymphocyte subsets, which provide a significant resource for clinical decisions and interpretation of immunological research in general, have been published in multiple populations throughout the world [1–8]. Existing normative data has several limitations including small sample sizes, incompletely described sample populations and flow cytometry techniques, and often limited depth regarding the immune cell populations described. Control data for immunologic studies are often obtained by performing assays of interest in small numbers of “healthy” volunteers, usually with little information on the volunteer’s demographics, medical history, immunization status, or other variables that may affect their immune system. Due to the small sample size, the estimates of immune system variables in these healthy controls may be imprecise, subject to large influence from “outliers”, and, in many cases, may not be representative of the overall population. Further compounding these issues are a lack of standardization in flow cytometry methodologies between laboratories, such as use of different reagents, markers, and gating strategies. These factors increase variability, confound comparisons among laboratories, and likely contribute to poor reproducibility of study results, ultimately limiting the usefulness of this powerful tool for investigations of the immune system.

To overcome these limitations and drive the field forward, leaders of the Human Immunology Project Consortium (HIPC) proposed standardized methods for immunophenotyping starting with flow cytometry [9]. In addition to improving the quality of flow cytometry in clinical trials and other immune system investigations, the long-term lofty goal of HIPC is to define the human immune system in health and disease, something only possible with rigorous standardization of flow cytometry methods [10]. HIPC proposed standardization in sample handling, reagents, instrument setup and data analysis. A central recommendation was a highly standardized 8-color flow cytometry assay for the identification of T cell, regulatory T cell (Treg), B cell, and innate cell (dendritic, natural killer [NK], and monocyte cells) subsets. Reference ranges for flow cytometry assays performed using these standards have not been published.

We combined the standardization of flow cytometry methods with the recruitment of very well-defined healthy controls that span multiple age groups, sex and race. Leveraging existing testing performed at Biomat USA (Grifols) plasma donation centers, subjects were normal source plasma donors who met all routine eligibility criteria and deemed healthy based on health history questionnaire, medical history, physical examination, and laboratory screening. In this study we define lymphocyte reference parameters for HIPC high-dimensional flow cytometry panels in the healthy human immune system. The HIPC phenotyping panels were first published in 2012 and, since that time, there has been increased interest in follicular helper T cells (Tfh), which was not captured within the HIPC phenotyping panel. Tfh cells are a subset of CD4 T cells vital to the generation of high-affinity memory B cells. Aberrant Tfh responses have been associated with autoimmune diseases, and in vaccine studies, Tfh cells are monitored as a potential marker of vaccine immunogenicity. Since Tfh cells were missing in the original Th subset panel, we included reference ranges for Tfh cells and its subsets from an internally developed 12-color T follicular helper cell (Tfh) panel constructed using common Tfh markers [11–13]. Though not a HIPC proposed panel, we developed a Tfh panel that will support ongoing research in the field and captures blood memory Tfh cells and follicular regulatory (Tfr) T cells, along with the characterization of activation state and Tfh subset [14, 15]. Our study also provides needed reference range information for African-American populations, which have been underrepresented in prior studies, and identifies differences in certain cell subsets between Caucasians and African-Americans that may have important clinical implications. Collectively, the entire dataset has been organized into an interactive website that provides our detailed methodology and can filter immunological data across age, sex, and race. Our goals for making these data available are to increase the standardization and reproducibility of flow cytometry assays, develop benchmarks for automated gating algorithms, and to provide normative frequencies from medically vetted healthy controls.

## Material and methods

### Enrollment and subject eligibility

Healthy volunteers eligible for normal source plasma donation at Biomat USA (Grifols) plasma donor centers in North Carolina and meeting study specific eligibility criteria were enrolled in the study. To assess eligibility for plasma donation a standardized screening process was performed. Screening consisted of a recent health history questionnaire, medical, surgical, social and travel history and physical examination, and completion of a questionnaire addressing high risk behaviors or potential exposures for plasma donation (e.g., high risk sexual behavior, blood transfusion, prion disease exposure). Subjects older than 66 are not eligible for plasma donation and were excluded from the study. Vital signs including blood pressure, pulse, temperature, and weight were taken. Study subjects must have a body mass index ≤35. Laboratory screening procedures included a hematocrit and total protein, urinalysis for glucose and protein, serum protein electrophoresis, nucleic acid tests for HIV, Hepatitis A, Hepatitis B, Hepatitis C and Parvovirus B-19, serology for HIV, Hepatitis A, Hepatitis B, Hepatitis C, rapid plasma reagin (RPR) test to screen for syphilis and an Indirect Coomb’s test. Subjects with no clinically significant laboratory values and otherwise meeting eligibility for plasma donation were approached for the study.

After written informed consent, subjects were administered a study specific questionnaire soliciting demographic data, medical history (including cancer and autoimmune disease), family history, and use of prescription or over the counter medications within the last 10 days. Subjects taking oral or injected immunosuppressants in the 6 months prior to enrollment or medications with anti-inflammatory properties were excluded from the study. The subject’s height and body mass index were recorded, and a urine pregnancy test was performed for females of childbearing potential.

At least 25 subjects were enrolled in each of the following age cohorts: 18–29 years, 30–39 years, 40–49 years, and 50–66. Cohorts were enrolled to ensure an approximately even gender distribution and preferably at least 20–30% non-Caucasians. The composition of each age cohort, in terms of gender and race, was monitored by the coordinating center at the Duke Clinical Research Institute. Clinical data was entered into a REDCap database [16]. This study was approved by the Duke local and Copernicus central IRBs.

### Blood collection and sample processing

Approximately 50mL of blood was collected from each subject in Acid Citrate Dextrose tubes (BD Vacutainer, Franklin Lake, NJ) and transported at room temperature to the Duke Immune Profiling Core (DIPC) processing and cryopreservation. Within 6 hours, mononuclear cells were separated by Ficoll density gradient centrifugation, washed and counted prior to storage. Cells were re-suspended in a 90% FBS (Gemini, West Sacramento, CA) and 10% DMSO (Sigma, St. Louis, MO) solution, and progressively cooled to ^-^80^0^ C in a CoolCell cell freezing container (BioCision, Larkspur, CA). The cells were transferred to liquid nitrogen for long-term storage on the following day.

### Flow cytometry

All flow cytometry assays were performed at the Duke Immune Profiling Core (DIPC). PBMCs were thawed following previously published methods [17] and counted following manufacturer procedures using a Muse cell analyzer and Muse Count and Viability Assay kit. One million viable peripheral blood mononuclear cells (PBMC) were surface stained for 45 minutes at ambient temperature with five pre-mixed, 8-color lyophilized HIPC panels obtained from BD Biosciences Custom Technology Team [9, 18] and a vital dye (Zombie Green or Aqua Fixable Viability Dye, BioLegend). For the Tfh panel, titrated amounts of each antibody was combined to make a staining cocktail that is used to distribute the same volume to each test. **Tables 1 and 2** describe a list of monoclonal antibodies, conjugates, and clones. After staining, cells were washed four times with PBS containing 0.5% fetal bovine serum, and resuspended in PBS containing 1% formaldehyde. Within 6 hours of staining 500,000 live cells were acquired for each sample using a calibrated BD Special Order Research Product 18-color LSRII analyzer. The HIPC lyoplates contained pre-stained compensation beads, used to set voltages to established Target Channels for each detector. FlowJo version 9.8.3 was used to analyze the resulting flow cytometry standard files and a gating template was applied for each respective panel. For each batch of experiments, a batch control was included in the HIPC plate to detect any batch-to-batch variations.

**Table 1:** Reagents and antibodies for HIPC panels.

Fluorochrome	T cell		Treg		B cell		DC/Mono/NK		Th1/Th2/Th17	
	*Marker*	*Clone*	*Marker*	*Clone*	*Marker*	*Clone*	*Marker*	*Clone*	*Marker*	*Clone*
**FITC**	Live/Dead	Live/Dead	Live/Dead	Live/Dead	Live/Dead	Live/Dead	Live/Dead	Live/Dead	Live/Dead	Live/Dead
**PE**	CD197	150503	CD25	2A3	CD24	ML5	CD56	B159	CD183	1C6/CXCR3
**PerCP-Cy5.5**	CD4	SK3	CD4	SK3	CD19	SJ25C1	CD123	7G3	CD4	SK3
**PE-Cy7**	CD45RA	L48	CD194	1G1	CD27	M-T271	CD11c	B-Ly6	CD196	11A9
**APC**	CD38	HIT2	CD127	HIL-7R-M21	CD38	HIT2	CD16	B73.1	CD38	HIT2
**APC-H7**	CD8	SK1	CD45RO	UCHL1	CD20	2H7	CD3+CD19+CD20	SK7+SJ25C1+2H7	CD45RO	UCHL1
**V450**	CD3	UCHT1	CD3	UCHT1	CD3	UCHT1	CD14	MϕP9	CD3	UCHT1
**BV510**	HLA-DR	G46-6	HLA-DR	G46-6	IgD	IA6-2	HLA-DR	G46-6	HLA-DR	G46-6

**Table 2:** Reagents and antibodies for Tfh panel.

Fluorochrome	Tfh	
	*Marker*	*Clone*
**PE**	CXCR3	IC6
**PE-Dazzle 594**	CCR7	G043H7
**PerCP-Cy5.5**	CD4	SK3
**FITC**	ICOS	C398.4A
**Alexa Fluor 647**	CXCR5	RF8B2
**Alexa Fluor 700**	CD45RA	HI100
**APC-Cy7**	CD3	SK7
**BV421**	CD25	2A3
**BV510**	CD8	SK1
**BV605**	CCR6	G034E3
**BV650**	CD127	A019D5
**BV711**	PD-1	EH12.2H7

**Fig 1:**
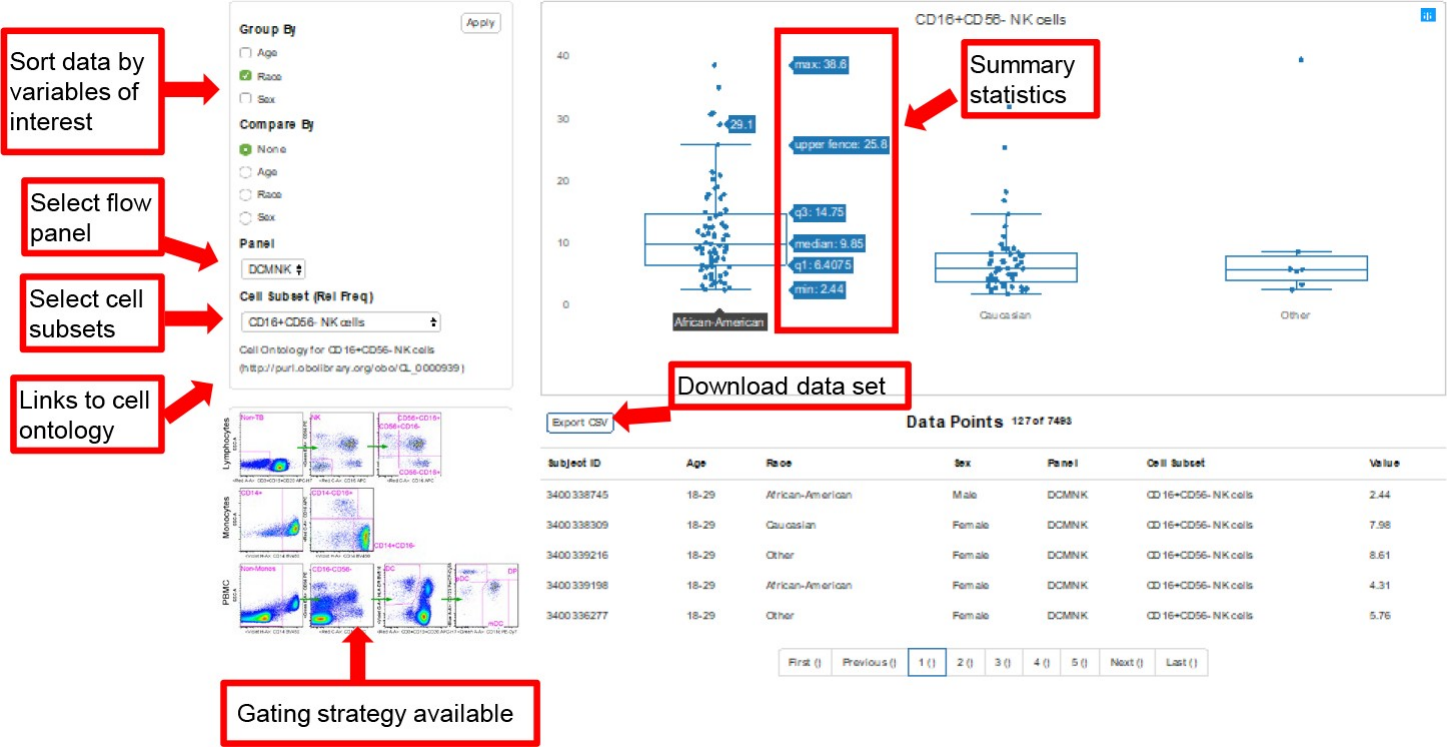
Example of web-based data visualization tool. Researchers have the ability to select reference ranges for flow cytometry panels of interest by age, gender, and race filters. This tool will calculate summary statistics on the selected population and the data may be downloaded for study. Links to cell ontology are present when applicable, and the gating strategies are available. Gating strategies and summary tables are also available.

The five panels are designed to measure activation and maturation across leukocyte subsets, including T-cells, B-cells, dendritic cells (DC), monocytes, and natural killer cells (NK) (**Fig 1**). Analysis gates were drawn as follows: time was used to exclude air bubbles and clogs that might have occurred during acquisition for all files. Zombie Fixable Viability dye was used to exclude non-viable cells, singlet gating was used to exclude aggregates, and scatter gates were drawn for lymphocytes, monocytes, and dendritic cells. In the T-cell panel, T cells (CD3+), T-helper (CD4+CD3+) and T-cytotoxic (CD8+CD3+) were measured off of lymphocytes, CD4+ and CD8+ maturation subsets were determined using CD197 (CCR7) and CD45RA and include naïve (CD197+CD45RA+), effector (CD197-CD45RA+), central memory (CD197+CD45RA-), and effector memory (CD197-CD45RA-). From each maturation subset, activation was defined as CD38+HLA-DR+. In the regulatory T cell (Treg) panel, CD4+CD3+CD25+CD127low were used to measure Tregs and CD194 (CCR4) with CD45RO identified memory Tregs. In the B-cell panel, B cells were identified as CD3-CD19+ lymphocytes. For B cell maturational subsets, T-cells (CD3+) were excluded before gating on CD19+CD20+ B-cells, then the following subsets were measured: naïve (CD27-IgD+), unswitched memory (CD27+IgD+), two populations of switched memory (CD27+IgD- and CD27-IgD-), and transitional B cells (CD38hiCD24+). From the B cell gate, two populations of plasmablasts were identified including, CD38hiCD27hi and CD38hiCD20low. In the DCMNK panel, [18] 3 populations of NK cells were defined as CD3- lymphocytes expressing the following combinations: CD16-CD56+, CD16+CD56+, and CD16+CD56-. Three populations of myeloid cells were measured: CD14+ monocytes, CD14+CD16- (conventional or M1) monocytes, CD14-CD16+ (non-conventional or M2) monocytes, and myeloid-derived suppressor sells (MDSCs, HLA-DRlo/-CD14+). Dendritic cells (DC) were defined as Lineage (CD3, CD19, CD20, CD16, CD56)- HLA-DR+) PBMCs. From the DC gate, plasmacytoid DC (pDC; CD123+CD11c-) and monocytoid DC (mDC; CD123-CD11c+) were also measured. Fluorescence minus one (FMO) controls were used to verify positive analysis regions.

### Statistics

The International Federation of Clinical Chemistry recommends a sample size of at least n = 120 for determination of laboratory reference values (International Federation of Clinical Chemistry. 1987, J Clinical Chemistry & Clinical Biochemistry). Based on this guideline, our study aimed collect samples from at least 120 healthy subjects evenly distributed across 4 age groups. A total of 127 healthy subjects completed the study, with at least 30 subjects in each age group. For the Tfh, Treg, B cell and innate panels, all 127 samples were analyzed, but due to technical issues, 123 samples were analyzed for the T cell panel.

Descriptive statistics, including means, standard deviations, standard error of the mean, medians, 25^th^ and 75^th^ percentiles, minimums, and maximums were generated for each panel and population. Where the same immune cell population was defined in multiple panels (lymphocytes, T cells, CD4+ T cells), we present the average value over these panels for that subset. Descriptive statistics are presented for the overall study population as well as by subgroups (age, gender, and race). In particular, age data are presented in the following pre-specified age ranges: 18–29 years, 30–39 years, 40–49 years, and 50–66 years. Race is presented as Caucasian/white or African-American/black. Due to the very low number of enrolled subjects identified as belonging to non-Caucasian or African-American racial groups (e.g., Asian), these subjects were combined into an “other” race category. We excluded “other” race for presentation in this manuscript due to the low numbers in this group, but all subjects are included in the online dataset.

The Clinical and Laboratory Standards Institute (CLSI) and the International Federation of Clinical Chemistry (IFCC) recommend that a non-parametric method is preferred when the number of reference individuals is at least equal to 120. Therefore, for the overall study population (N = 127), we performed a non-parametric bootstrap to calculate reference intervals (percentile 2.5–97.5). Non-parametric analysis requires no assumptions about the underlying distribution of the data. Non-parametric bootstrap estimates were calculated similarly to the rank-based method. A bootstrap sample was generated, and the data was sorted by increasing numerical values and assigned ranks. We computed the lower and upper empirical percentiles, with linear interpolation as necessary. We then further used bootstrapping to construct the 90% confidence intervals around the lower and upper percentiles using the percentile interval method.

Since the overall goal of the project was to provide reference ranges for flow cytometry assays, we performed testing for statistical significance only to highlight certain data in the manuscript. If visual inspection of the summarized data suggested a difference between a particular subgroup may be present, we performed a two-sample t-test when data were normally distributed. When data were not normally distributed, we performed a non-parametric Wilcoxon rank-sum test. Level of statistical significance was set at P<0.05. We performed no corrections for multiple comparisons.

### Web-based data visualization tool

Comprehensive data and methods are available on a freely accessible website: https://duke-hhis.github.io/reference-range. The website is supported by Internet Explorer 10 (and above), Microsoft Edge, as well as recent versions of Mozilla Firefox and Google Chrome. Method related information on the website include the flow cytometry reagents and gating strategies. Summary statistics and reference ranges for major cell subsets are available in tabular form for the overall study population and by race, age, and gender. Links to cell ontology are available for certain immune cell subsets. For additional analyses not presented in tabular form, investigators can use the web tool to select the sample population of interest, the flow cytometry panel, and the cell subset. Once these variables are selected, the web tool will present the reference ranges for the sample visually with summary statistics and calculated reference ranges with confidence intervals (**Fig 1**). The de-identified dataset for a sample population of interest may be downloaded as a CSV file.

## Results

### Enrollment

Following the informed consent process, subjects underwent a rigorous screening process consisting of medical, surgical, social and travel history, physical examination, questionnaire addressing high-risk behaviors or potential exposures, current medications/ vitamins/ supplements, vital signs, and family history. Laboratory testing was performed, which included blood chemistry, serum protein electrophoresis, atypical antibody screen, blood counts, infection screen (HIV, Hepatitis B, Hepatitis C, Hepatitis A and Parvo B-19, syphilis), and a pregnancy test for females. A total of 193 subjects consented for the study. There were 22 screen failures, and blood samples were collected from 166 subjects (**S1 Fig**). Flow cytometry assays were performed on samples from 127 subjects (analysis population).

### Demographics

In the overall analysis population there was balance in the number of subjects included in each age cohort and a near even distribution of males and females (**Table 3**). Within each age cohort, the distribution by sex was also approximately 50% (male: 18–29 51.7%; 30–39 54.0%; 40–49 50.0%; 50–66 53.4%). Subjects self-reported race by selecting from an established panel of descriptors, and African-American subjects represented about half of the analyzed samples and the remainder were predominantly Caucasian. No subjects had a history of cancer or autoimmune disease, and a reported family history of autoimmune disease was present in 10 subjects (7.9%). No subjects had a personal or family history of primary immune deficiency. Only one subject had a vaccination within the 60 days prior to enrollment (influenza, 21 days prior to enrollment). Ninety-five percent of subjects (n = 120) had not taken any prescription or over the counter medication within 10 days of their blood draw.

**Table 3:** Study population demographics.

	Total (N = 127)
**Age (years)**	
Mean (SD)	39 (12.3)
Median	39
Q1, Q3	29, 50
Min, Max	18, 64
**Age cohorts (years)**	
18–29	32 (25.2%)
30–39	33 (26.0%)
40–49	30 (23.6%)
50–66	32 (25.2%)
**Sex**	
Male	65 (51.2%)
**Race**	
Caucasian	51 (40.2%)
Black or African American	69 (54.3%)
Other	7 (5.5%)
**BMI (kg/m2)**	
Mean (SD)	26 (4.0)
Median	26
Q1, Q3	23, 30
Min, Max	18, 35

### Adverse events

Mild adverse events occurred in 2 subjects (hematoma, n = 2 and vein infiltration, n = 1). There were no serious adverse events.

### Online data

In order to organize the large dataset and allow for flexible and quick visualization of the results, a web page was developed to share the methods, reference ranges and data generated by this study (https://duke-hhis.github.io/reference-range). The website is supported by Internet Explorer 10 (and above), Microsoft Edge, as well as recent versions of Mozilla Firefox, Safari, and Google Chrome. Researchers can filter the entire dataset by variables of interest such as flow cytometry panel, lymphocyte subset, gender, age, and race to select a healthy reference population (**Fig 1**). The web resource will then calculate summary statistics on the selected population and the filtered dataset may be downloaded for study. Links to cell ontology are present when applicable. Rather than attempting to comprehensively summarize data from the extensive available dataset, the following sections present a high-level overview and highlight selected findings.

### Overview of flow cytometry panels

**Fig 2:**
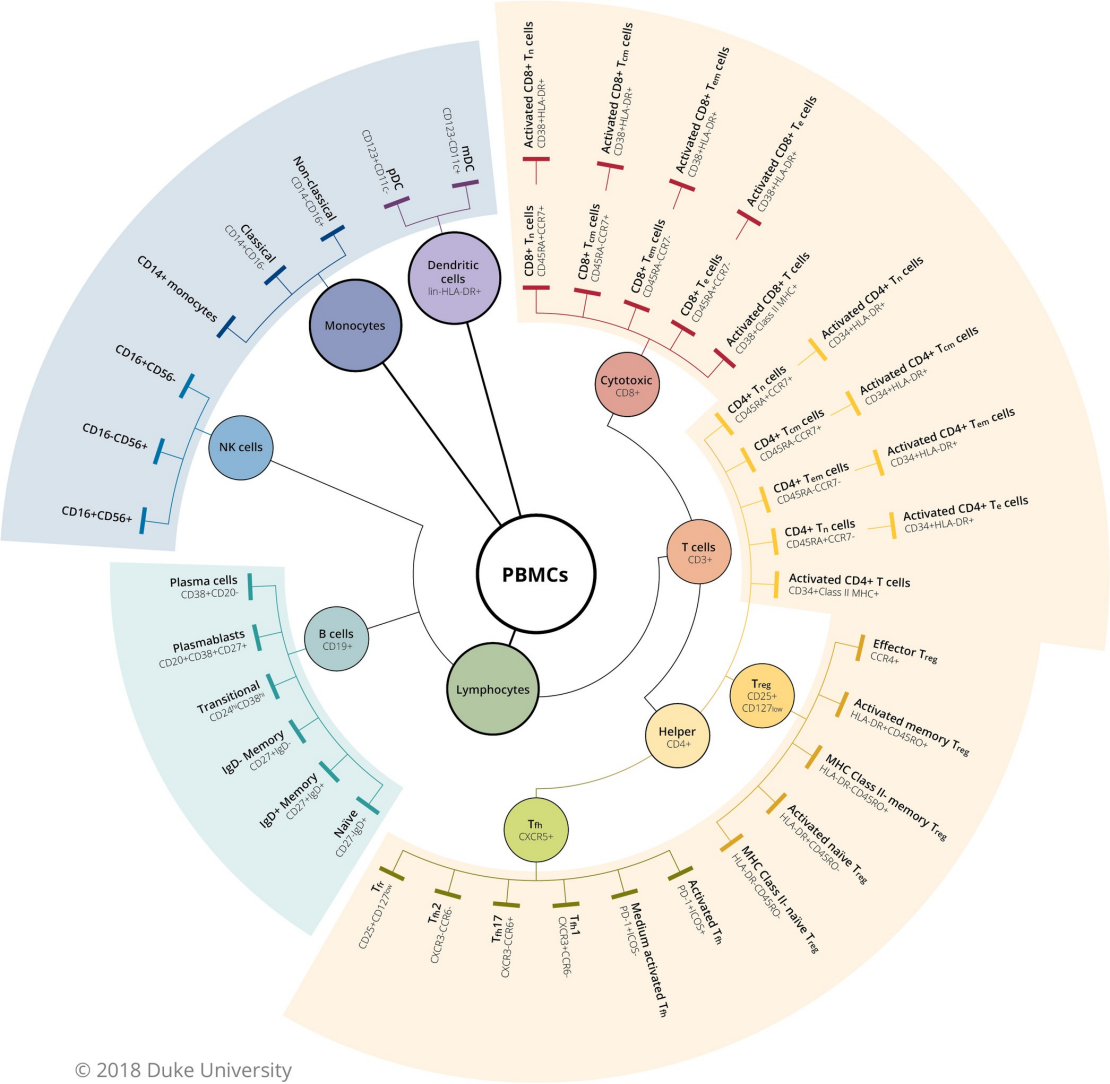
Identification of cell subsets in the blood. T, B, and innate cells were identified according to HIPC guidelines. The Tfh panel is not part of the HIPC guidelines and markers were selected by the study team based on published medical literature.

**Fig 3:**
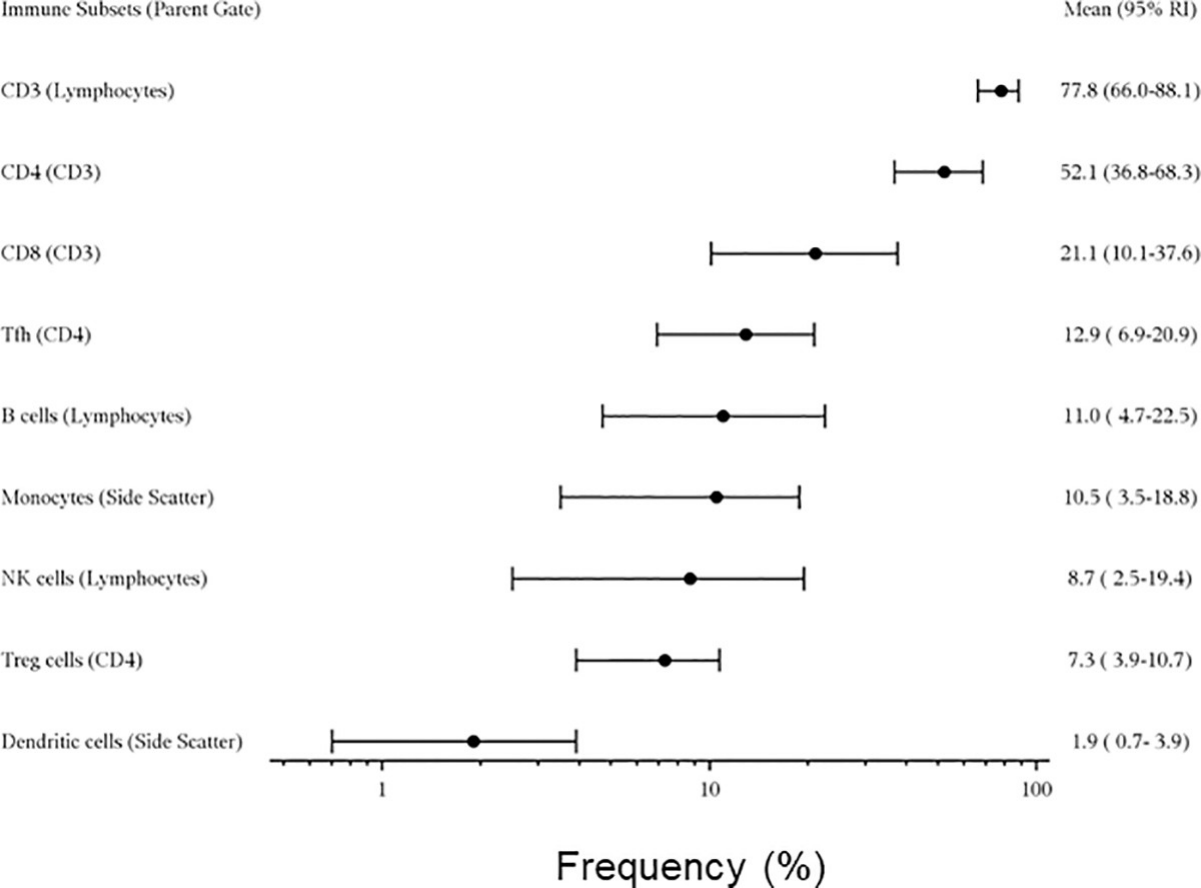
Forest plot summarizing reference ranges for major lymphocyte subsets in the overall population. References are shown for CD3+ T cells, CD4+ T cells, CD8+ T cells, Tfh cells, overall B cells, monocytes, NK cells, Tregs, and dendritic cells. Data are presented as means with 95% reference intervals calculated by non-parametric bootstrap.

**Fig 4:**
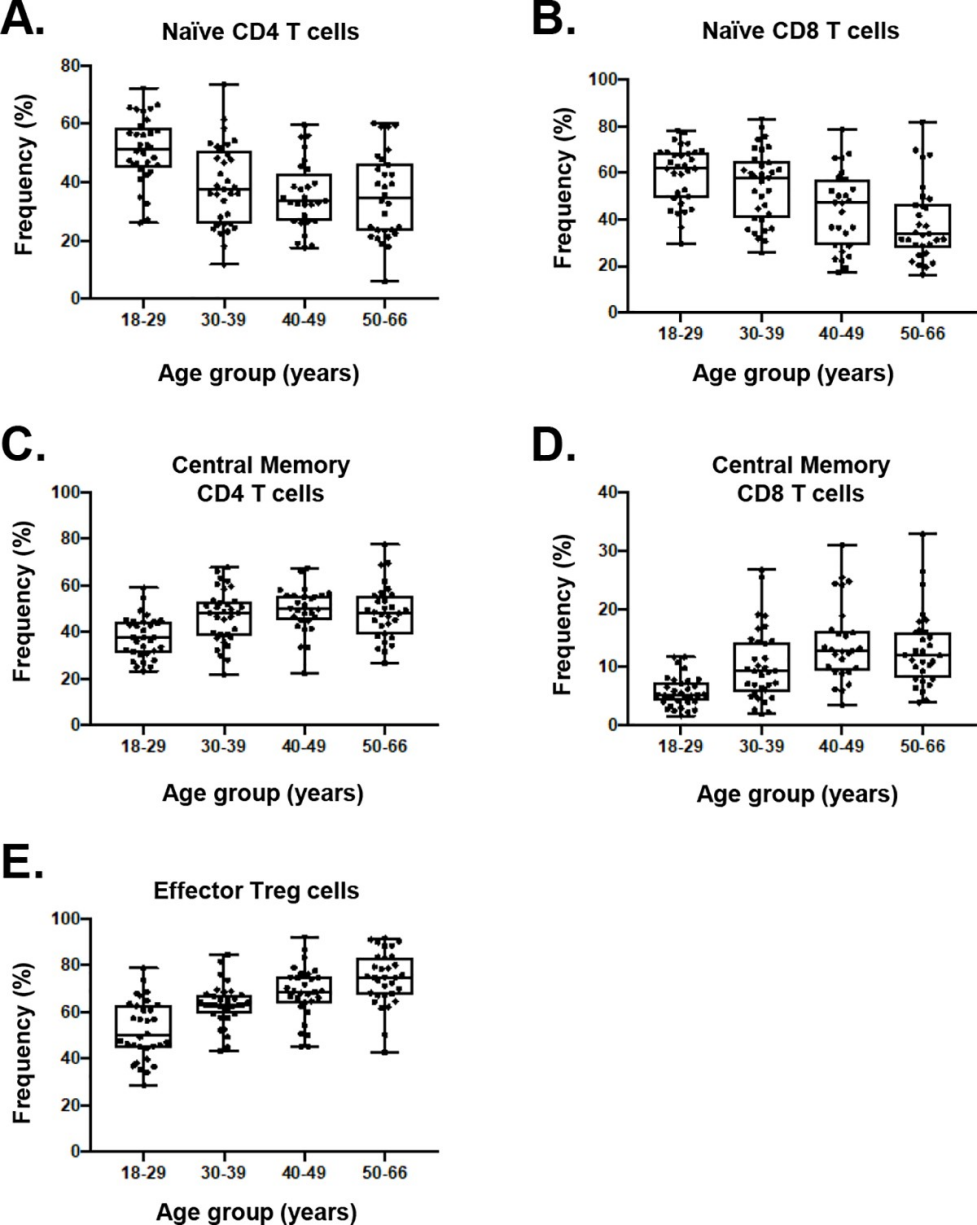
Age related changes in selected T cell populations. A, B) A decrease in naïve CD4+ and CD8+ T cells with advancing age (C, D) corresponds with an increase in central memory CD4+ and CD8+ T cells. E) Effector memory CCR4+ Tregs increase with age. Tregs, identified by gating on CD4^+^CD25^+^CD127^low^ cells were compared based on four age groups.

Polychromatic flow cytometry assays were completed for T cell (N = 123; 4 samples failed QC), Tfh cell (N = 127), Treg cell (N = 127), B cell (N = 127), and innate cell panels (N = 127) that in total identify greater than 50 distinct immune cell subsets (**Fig 2**). The T cell, B cell, and innate cell panels were completed according to HIPC guidelines (**Table 2**) [9] Cell subset identification of Tfh cells has not been defined by HIPC. Therefore, we developed a twelve-color panel that provides reference range data for Tfh populations (**Table 3**). Reference ranges for major lymphocyte subsets in the overall population are shown in **Fig 3**. Reference range tables calculated from a non-parametric bootstrap (percentile 2.5–97.5) with 90% confidence intervals for lymphocyte subsets are available on the website and presented for the overall study population, as well as by age, gender, and race. Major sex differences were not observed in any of the panels or by age group. Overall, expected age-related changes were observed in several cell subsets. For example, frequencies of naïve CD4+ and CD8+ cells decreased with age, while CD4+ and CD8+ memory T cell populations increased with age (**Fig 4A–4D)**.

#### Treg panel

The frequencies of CD4+CD25+CD127low Tregs were similar by sex and remained relatively stable with age. African-Americans had higher mean overall Treg (7.7 standard deviation +/-1.7 vs. 6.7+/-1.8, p = 0.0017) and peripheral CCR4+ effector memory Tregs frequencies than Caucasians (67.2+/-12.5 vs. 61.8+/-14.4, p = 0.0292). Peripheral CCR4+ effector memory Tregs increased with age including among all racial groups (**Fig 4E**).

#### Tfh panel

**Fig 5:**
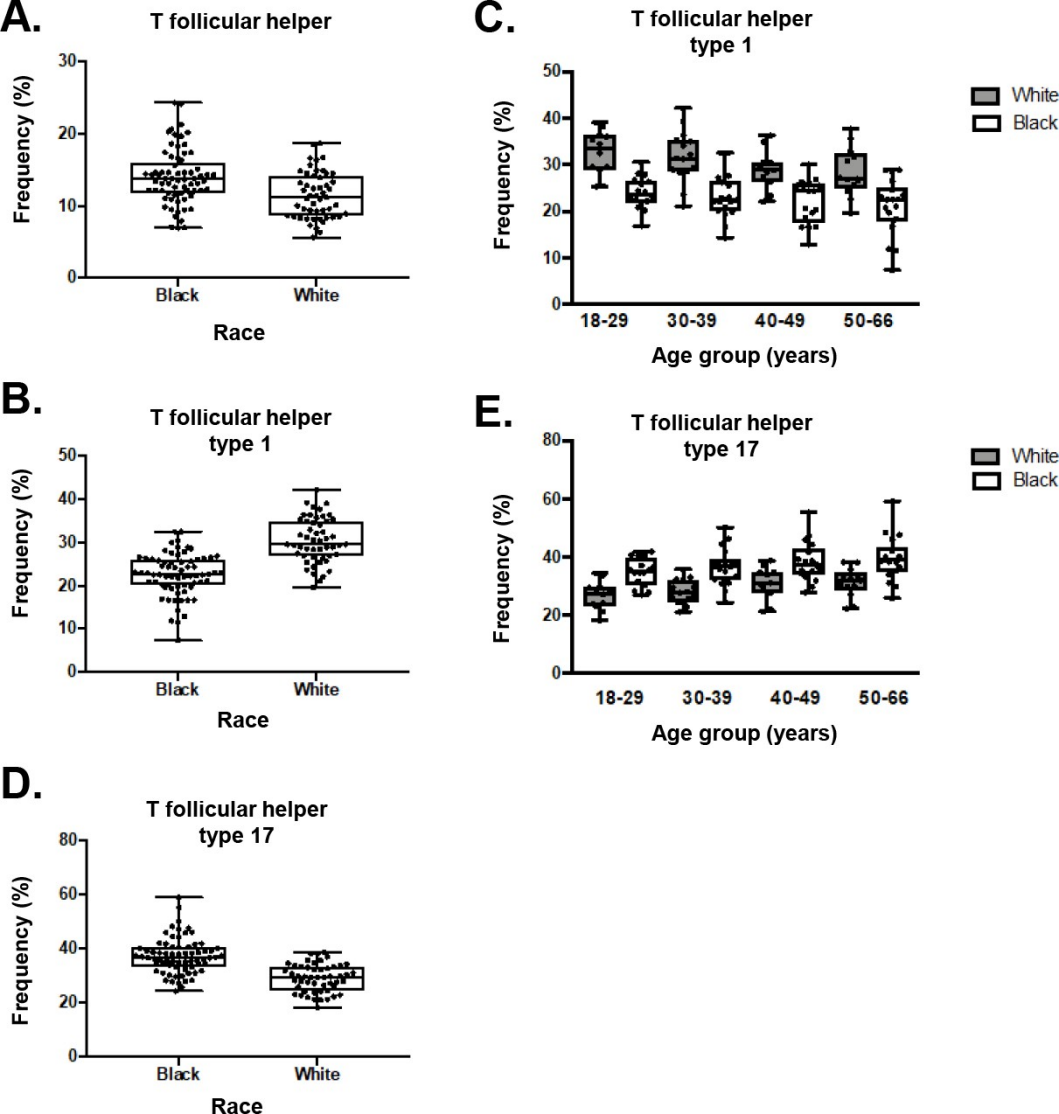
Racial disparities in the distribution of Tfh cells. A) Overall Tfh frequencies were similar between African-Americans and Caucasians. However, race related differences were observed in certain Tfh cell subsets including B, C) decreased frequencies of Tfh1 cells in African-Americans at all age ranges, and D, E) increased Tfh17 frequencies in African-Americans, a difference that was observed in all age groups. In C and E, Caucasians are represented by blue bars and African-Americans by orange bars.

The Tfh panel was developed to complement the profiling of CD4 T cells and other T helper subsets in the HIPC panel. The combination of the 12 markers that make up this panel identifies Tfh cells through the combination of CXCR5 and CD45RA expression, and activation status through PD-1 and ICOS. African Americans had higher frequencies of Tfh cells compared to Caucasians (14.0+/-3.8 vs. 11.4+/-3.2, p = 0.0005) (**Fig 5A**). Tfh1, Tfh2, and Tfh17 cells are Tfh subsets that differ in their capacity to help B cells become Ig producing cells [14, 15, 19, 20]. Of the three, Tfh17 cells (CXCR3-CCR6+) are the most efficient at supporting antibody producing B cells. A more in-depth analysis of Tfh cell subsets reveal a disparity between African-Americans and Caucasians, where African-Americans had a lower frequency of Tfh1 cells (22.6+/-4.9 vs. 30.4+/-5.2, p<0.0001) and a higher frequency of Tfh17 cells (37.4+/-6.6 vs. 29.2+/-5.1, p>0.0001) **(Fig 5B and 5D**). This difference in Tfh1 and Tfh17 cells was observed across the four age groups (**Fig 5C and 5E**).

#### Innate cell panel

**Fig 6:**
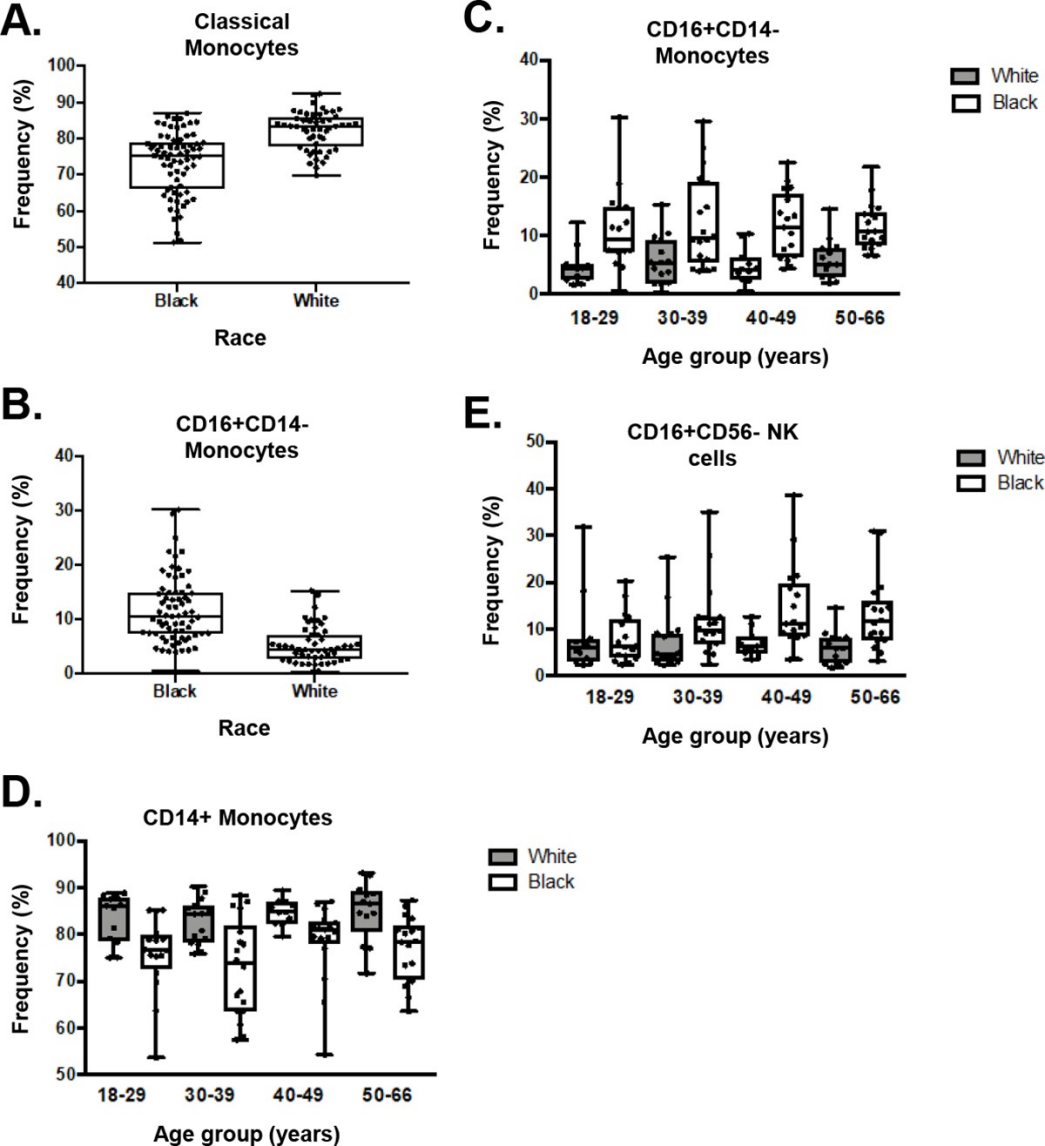
Racial disparities in the distribution of innate cells. A) Classical (CD14+CD16-) monocytes were decreased and B) non-classical (CD16+CD14-) were increased in African-Americans compared with Caucasians. C) The increase in CD16+CD14- populations in African-Americans was present in all age groups. D) In each age group, the frequency of CD14+ monocytes was higher in Caucasians compared to African-Americans. E) CD16+CD56- NK cell frequencies were increased in African-Americans compared to Caucasians, and the difference became larger in the older age groups. In C, D, and E, Caucasians are represented by blue bars and African-Americans by orange bars.

The innate cell panel includes HIPC markers for monocytes, dendritic, and NK cells (**Fig 2**) and allows for identification of additional innate cell subsets. We found that CD14+ monocyte and classical monocyte (CD14+CD16-) frequencies were decreased in African-Americans compared to Caucasians (CD14+ monocyte: 76.0+/-8.8 vs. 83.9+/-4.9, p<0.0001; classical monocyte: 73.3+/-8.8 vs. 82.2+/-5.3, p<0.0001) (**Fig 6A and 6D**), while non-classical monocytes (CD16+CD14-) were increased (11.7+/-6.2 vs. 5.4+/-3.4, p<0.0001) (**Fig 6B and 6C**). Among NK cells, we observed increased CD16+CD56- NK cells in African-Americans (11.9+/-7.9 vs. 7.3+/-5.7, p<0.0001), a difference that appeared to increase with advancing age (**Fig 6E**). Other innate subsets were relatively similar according to race and sex.

## Discussion

In this study we defined reference range standards for highly standardized polychromatic flow cytometry assays for major immune cell subpopulations in a very well-characterized healthy sample population from normal source plasma donors according to HIPC recommendations. A distinct strength of our study was the rigorous screening of subjects for active infections, immune conditions, exposures, and medications that could potentially disturb the normative data we wanted to collect. We efficiently performed this screening by leveraging the existing screening process required for normal source plasma donors at Biomat USA (Grifols) donor centers. This screening process ensured that the study population was indeed as healthy as possible and was more rigorous than is typically performed in most immunology studies investigating disease populations or even studies establishing reference ranges.

The complexity of flow cytometry assays has increased significantly, but efforts to improve standardization and provide appropriate normative data for reference ranges have not kept pace. In many cases, small sample sizes, uncertainties about the composition of the study population, and a lack of detail in the flow cytometry methodology (e.g., gating strategy and reagents) limit the utility of existing reference ranges. As a result, standardization of flow cytometry remains a major challenge to the accurate description of the healthy and diseased human immune system [9]. Since much of the variance in flow cytometry assays is related to technical differences between laboratories and operators [21], the sample processing and storage, reagents, instrumentation, and analysis strategy from our study are freely available. This transparency can lead to technical standardization resulting in more consistent flow cytometry results, an enhanced ability to compare results across studies, and lead the field closer to the HIPC’s stated goals to 1) “define profiles/signatures/fingerprints of steady-state and activated human immune system” and 2) “create centralized knowledge base and resource” [22].

Data from this study will benefit several additional aspects of immunological research. A particular strength of our sample population is the high representation of African-Americans, who have often been underrepresented in prior efforts to define flow cytometry reference ranges in the United States or the race of the sample population has not been fully documented [23]. In addition, we propose a gating strategy that can be standardized across studies, and we have provided our data in two formats to aid investigators. We developed a web tool for investigators interested in using these healthy reference ranges in their research. Using this web tool, populations may be identified based on predefined criteria, such as age, sex, or race, and the values can be downloaded in a CSV file for further statistical analysis. Along these lines, we also envision that these reference data and methods could be a valuable training resource for individuals learning flow cytometry and for use in multicenter collaborations to maintain consistency among sites. Trainees can compare their results obtained on their own training samples with the gating strategy and results from our study.

As is commonly encountered in flow cytometry studies, comparisons of our data with prior studies are difficult due to varying methodologies and patient populations [24, 25]. A recent publication by Zalocusky et. al. undertook a large bioinformatics project to isolate data of healthy normal human subjects from the ImmPort database, and integrate the data for visualization [25]. They were able to capture 10,344 subjects from 83 studies, and normalized and integrated flow cytometry, protein, and gene expression datasets. Since the data was generated from 83 studies, it remains unknown whether a lack of oversight may have caused mistakes in labeling or data description. In addition, the screening process for determining whether the healthy controls from all 83 studies were indeed healthy was variable. Our study complements the 10,000 Immunomes Project by providing healthy flow cytometry data in areas underrepresented in that dataset including subjects 40–50 years of age, Black/African-Americans, and innate cell populations. In another study of immune subsets in healthy people, Patin et. al conducted a large-scale study of 1000 healthy volunteers of western European ancestry who were carefully screened. However, the volunteers were all descendants of mainland French persons for at least 3 generations [26]. Their study was focused on intrinsic, environmental and genetic factors that affect immune cells [24].

A novel contribution of our data is the addition of a 12-color Tfh panel performed according to HIPC methodologies for evaluation of T cell subsets. Tfh cells, which were not included in the original HIPC panel, are a Th subset vital for the generation of high affinity memory B cells. Tfh cells are also implicated in autoimmune disease pathogenesis and vaccine immunogenicity. Thus, we included a Tfh panel to complement the subsets identified by the HIPC phenotyping panel. The Tfh panel identifies both Tfh cells and Tfr cells, and markers such as ICOS and PD-1 distinguish Tfh cells by their activation status. Chemokine receptors CXCR3 and CCR6 further discriminate Tfh cells into Tfh1, Tfh2, and Tfh17 subsets which differ in their capacity to help B cells [15]. Tfh2 and Tfh17 cells are efficient at driving naïve B cells to produce immunoglobulins and induce isotype switching through IL-21 secretion, whereas Tfh1 cells lack the capacity to help B cells [27]. The growing importance of Tfh cells in immunological research required that we provide a standardized methodology to identify and characterize this subset. With the advancement of flow cytometry platforms that expands the number of fluorescent parameters, a 12-color panel is not as challenging as it once was and can be performed at many research institutions with a flow cytometry core facility.

The Tfh panel revealed racial differences in circulating Tfh cell subsets with African-Americans demonstrating decreased frequencies of Tfh1 cells and increased frequencies of Tfh17 cells. Among Tfh cells, the Tfh17 subset provides very efficient helper capacity for B cells [15]. Previous studies have consistently shown that African-Americans, in comparison with Caucasians, demonstrate increased antibody responses to vaccines, including rubella, [28] pertussis [29], measles [30], and influenza [31]. Furthermore, racial differences in the prevalence of certain autoimmune diseases have been reported between African-Americans and Caucasians. For example, it is known that systemic lupus erythematous is more common and severe in African-Americans than Caucasians [32]. Interestingly, Tfh17 cell frequencies are increased in systemic lupus erythematous, particularly in active disease [33]. Based on our data, it is interesting to speculate that an underlying enhanced Tfh17 cell reactivity in African-Americans may at least in part underlie these observations related to vaccination and susceptibility to autoimmune disease and future research could further explore this possibility.

Our data are consistent with known effects of aging including fewer naïve CD4+ and CD8+ T cells and accumulation of T cell subsets with a memory phenotype [34–37]. This shift is thought to result from a gradual reduction of naïve T cells by the thymus with aging and thymic involution and increased immunologic memory as a result of exposure to environmental antigens and pathogens. The effect of aging on T cell frequencies has been described for some subsets. For example, increases in Treg cell populations (CD4+CD25+, CD4+FOXP3+) are well described [38–41]. In this study, there were no significant shifts in CD4+CD25+CD127low Treg frequencies with age. However, effector memory Tregs expressing CCR4+ increased with age, including among all racial groups [42]. A change in memory Treg frequencies has been hypothesized to contribute to an overall decrease in adaptive immune system reactivity among the elderly leading to increased risk of cancer and a reduced ability to clear infections [40, 42]. Compared with prior studies, the absence of an overall increase in Treg frequencies with age may relate to the study population, which did not include subjects over the age of 66 years, and used different markers to identify Tregs.

It has long been known that people of African ancestry tend to have lower white blood cell counts and that reduction is largely attributable to granulocyte populations, particularly neutrophils [43–45]. However, detailed descriptions of innate cell subsets with flow cytometry in healthy humans are mainly limited to overall NK cells where divergent findings according to age and gender have been reported in a predominantly Caucasian population [3, 23, 46–48]. Consistent with one of the few studies to consider racial differences in innate cell reference ranges by Appleby et al. [49], we found reduced frequencies of classical monocytes in African-Americans compared to Caucasians, in addition to increased CD16+CD56- NK cells. The Appleby study compared monocyte populations in Africans from an urban and rural environments with a local Caucasian cohort descended from Western Europeans and suggested that some differences in monocyte frequencies may be attributable to environmental exposures such as parasites. Since subjects from our study were drawn from the same geographic area and presumably had similar environmental exposures, it suggests that environment cannot completely explain the alteration in classical monocytes. Overall our data provide further support for considering racial differences when conducting and reporting immunological research in addition to nonimmune factors (e.g., socioeconomic and pharmacogenomics), as well as when interpreting racial differences in response to treatments, vaccination, and outcomes such as graft survival in transplantation [31, 50].

This study has limitations. Due to the constraints of routine eligibility criteria for normal source plasma donors, the upper limit of participation is 66 years old, we do not have data on older populations. For similar reasons, we do not have data in the pediatric population. The geographical region where the study was conducted has a relatively small population of people of Asian descent and, thus, they are underrepresented in this sample. Comparisons of fresh whole blood with cryopreserved PBMCs were not performed as part of this study, however, our own unpublished internal data on similar panels and those published in the medical literature support that minimal differences would be expected in the phenotypic markers in this study [51–53]. Finally, we did not directly address genetic factors that could affect immune reactivity and underlie some of the racial differences observed in our study [54–56]; rather we focused on generation of the reference ranges.

## Conclusion

The purpose of this study was to provide the human immunology community with a freely available resource for lymphocyte reference ranges, including novel reference ranges for certain immune subsets established using highly standardized and transparent methodologies from a rigorously described healthy human population. These data can serve as a reference point for future studies investigating perturbations of the human immune system such as vaccination, inflammatory and autoimmune diseases, and therapeutics that act on immune mediators and pathways. Further exploration of the existing dataset will likely lead to additional novel insights on the healthy human immune system and advance the HIPC mission.

## Supporting information

S1 Fig Consort diagram. (TIF)
