# Supplementary figures and images for: Establishment of normative ranges of the healthy human immune system with comprehensive polychromatic flow cytometry profiling
Source: PLoS One. 2019 Dec 11;14(12):e0225512. doi: 10.1371/journal.pone.0225512 (PMC6905525; doi:10.1371/journal.pone.0225512)

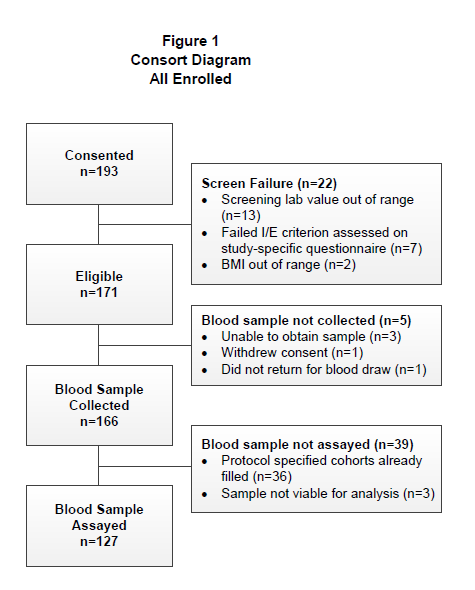

Supplement: S1 Fig — (TIF) [file pone.0225512.s001.tif]
